# Study on In-Service Inspection of Nuclear Fuel Assembly Failure Using Ultrasonic Plate Wave

**DOI:** 10.3390/s22197606

**Published:** 2022-10-07

**Authors:** Xiang Xiao, Guo Zheng Zhou, Ke Qing Wang, Feng Xi, Kun Zeng

**Affiliations:** 1 School of Artificial Intelligence, Chong Qing Technology and Business University, Chongqing 400067, China; 2 SWS Hemodialysis Care Co., Ltd., Chongqing 401123, China; 3 Suzhou Nuclear Power Research Institute, Suzhou 215004, China; 4 School of Automation, Wuxi University, Wuxi 214105, China; 5 DongFang Boiler Co., Ltd., Deyang 618000, China

**Keywords:** ultrasonic plate wave, nuclear failed rod, in-service inspection

## Abstract

As protection for nuclear power plants is quite necessary, the nuclear fuel is sealed in zirconium alloy thin wall cladding. During service, fuel rods might be damaged caused by wall-thickness thinning, cladding corrosion and cracking, etc. This will cause the coolant to enter into the fuel rod, which may lead to the failure of the fuel assembly. However, current diagnostic methods have limitations due to the special structure of the fuel assembly and the underwater and radioactive environment. In this paper, a novel inspection method is proposed to recognize the failure of a fuel rod. The fuel rod failure can be detected based on the presence or absence of coolant inside the fuel rod by using an ultrasonic plate wave. The inspection model and process algorithm are proposed for in-service inspection. The relationship between signal and scanning position is established and analyzed. Both ultrasound field simulation and experiment have been carried out for validation. The corresponding results illustrate that the failed nuclear fuel rod of the whole fuel assembly (including the internal rods) can be effectively detected without the influence of the near-field region by using the proposed method.

## 1. Introduction

Damage to fuel assemblies directly affects the safe operation of nuclear reactors. During service, fuel rods might be damaged by wall-thickness thinning, cladding corrosion and cracking, etc. When the fuel rod is broken, the fission products will leak out, and the coolant will enter the fuel rod at the same time. It is called the failure of a fuel rod. This will increase the probability of nuclear leakage accidents [1]. Fuel failure issues have significant operational impacts on nuclear power plants, including operating cost, radiation exposure, and plant availability [2,3].

Therefore, NDT methods have been developed to detect the failure of fuel rods based on the integrality of the metal material. Sipping is the most common technique used to test failed FAs in PWR [4]. The radioactivity is measured and analyzed using the sipping method to detect the existence of fission products to recognize fuel leakage [5]. It is able to measure fuel assembly damage economically and reliably without the disassembly of FA. However, sipping cannot determine the location of leaking fuel rods in the FA. In recent years, non-destructive testing (NDT) methods have been developed to measure the fuel rods. The IR thermography method was used to detect wall-thinning defects [6]. However, it is not suitable for ISI due to the underwater environment. Visual testing (VT) can be used to inspect for surface inspection under the water [7]. However, the internal fuel rods inside the FA cannot be inspected. Additionally, the precision of measurement will be affected by the thermal flow due to nuclear decay. The eddy current (EC) NDT method is another technology to inspect metal tubes in nuclear industry [8]. One of the EC techniques, called remote field eddy current (RFEC), installs the testing system to measure the gap in tube-in-tube systems [9]. Gros developed a new type of eddy current sensor for the inspection of heat exchange tubes [10]. There are rare reports that eddy current testing can be used to detect fuel rod failure. The eddy current testing technology is usually used to measure the thickness of oxide film on FA. Ultrasound testing (UT) is another NDT method widely used in nuclear power plants. The ultrasonic pulse–echo method has been applied to detect FA failure [11]. However, it is difficult to maintain the same geometry of beam alignment, which affects reliability during scanning [12,13]. An ultrasonic pulse-echo system for failed rods inspection was reported by Thome [3]. A sound transmission layer is added between the PVDF element and the rod. However, the space between neighbor rods is only 3.1 mm. Therefore, it is not suitable for ISI due to the deformation of the rods.

Under in-service inspection conditions, it is not easy to keep a distance of more than 1 mm during scanning due to the distortion of the fuel rod and the vibration of the mechanism. Furthermore, the probe center frequency was up to 25 MHz, which means that the requirements for the bandwidth and signal processing capacity of the instrument are high. Therefore, the cost is high.

Based on our previous work on zirconium alloy tubes [14,15] and Thome’s work [3], the fuel rod failure inspection method was proposed by using ultrasonic plate waves in this paper. The signal of the scanning process model is analyzed and the relationship between the signal and the scanning position is established. In addition, an ultrasonic inspection system for ISI FA’s failure is carried out. It enables (1) avoiding the influence of ultrasonic near-field region for ISI; (2) not relying on the high-performance instrument with wide bandwidth; (3) and the inspection of all the fuel rods in FA and locate the failed rod. 

The rest of the paper is organized as follows: Section 2 discusses the principle of failure measurement by using proposed ultrasonic plate wave method. Specifically, the signal of scanning process model is analyzed and relationship between signal with scanning position is established. Section 3 describes the experimental setup and Section 4 presents the experimental results and discussions. Finally, Section 5 concludes the works. 

## 2. Methodology

### 2.1. Introduction of Ultrasonic Plate Wave 

According to the characteristics of the plate wave, the ultrasonic wave in the tube can be divided into the symmetric mode and the antisymmetric mode. Each mode has a different order, usually represented by S_0_, S_1_, S_2_…A_0_, A_1_, A_2_…. Under the free boundary condition, the lamb wave frequency equation can be expressed as [16]:

For symmetrical modes:

(1) $$\frac{\tan h\left(\frac{\alpha T}{2}\right)}{\tan h\left(\frac{\beta T}{2}\right)}=\frac{{\left(k^{2}+\beta^{2}\right)}^{2}}{4k^{2}\alpha \beta }$$

where:

$$\alpha =k\sqrt{1-\frac{c_{ph}{}^{2}}{c_{l}{}^{2}}}\;\beta =k\sqrt{1-\frac{c_{ph}{}^{2}}{c_{s}{}^{2}}}$$

For the antisymmetric modes:

(2) $$\frac{\tan h\left(\frac{\alpha T}{2}\right)}{\tan h\left(\frac{\beta T}{2}\right)}=\frac{4k^{2}\alpha \beta }{\left(k^{2}+\beta^{2}\right)}$$

where *T* is thickness of the rod,$\;c_{ph}$ is Lamb wave phase velocity;$\;c_{l}$ is the longitudinal wave velocity; $c_{s}$ is the shear wave velocity and k is wavenumber.

The wavenumber can be obtained as:

(3) $$k=2\pi \frac{f}{c_{l}}$$

where *f* is the transducer center frequency.

The group velocity can be calculated from the corresponding phase velocity by using the slope formula.

(4) $$c_{gr}=c_{ph}\left[1-\frac{1}{1-\left(fT\right)\frac{dc_{ph}}{d\left(fT\right)}}\right]$$

where $c_{gr}$ is group velocity.

**Figure 1:**
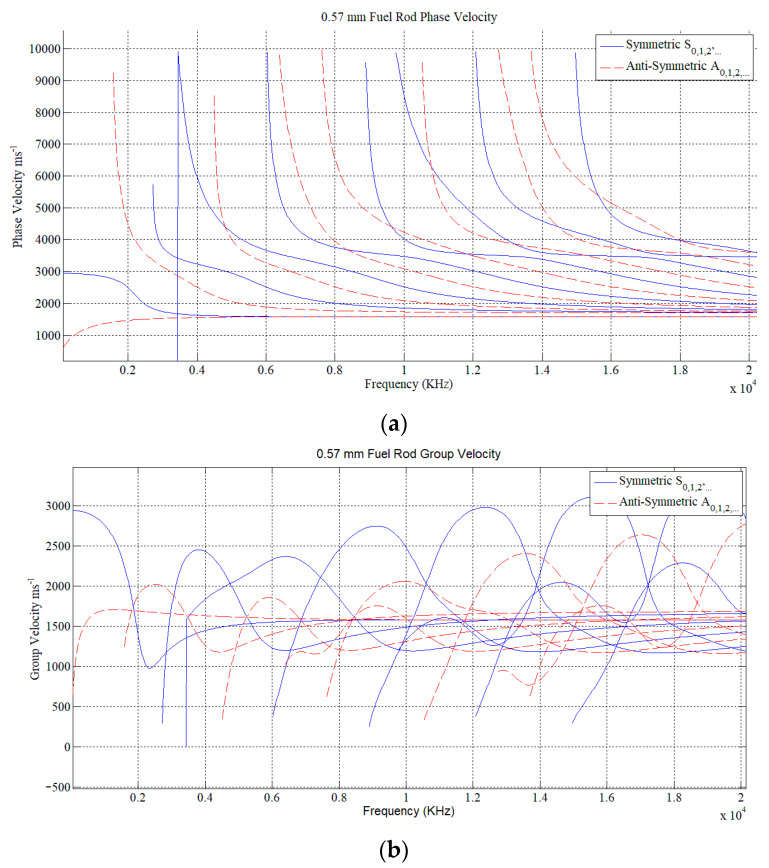
(**a**) the phase velocity; (**b**) the group velocity.

Thus, according to Equations (1)~(4) and the known ultrasonic velocity of zirconium alloy ($c_{l}$ = 4686 m/s,$\;c_{s}$ = 2360 m/s), the dispersion curve for fuel rod with thickness of 0.57 mm can be acquired as shown in Figure 1.

The mode of the plate wave can be selected according to the frequency equation or dispersion curve. The phase velocity of the wave mode can be obtained by the given plate thickness and the selected frequency of the transducer. In addition, the necessary condition for Lamb wave excitation is that the incident angle θ must meet the condition of:

(5) $$\theta =arcsin\frac{c_{l}}{c_{p}}$$

### 2.2. The Proposed Inspection Method

According to Thome’s work [3], the transducer should remain 1 mm apart from the rod face to avoid the influence of the near-field region. However, it is not suitable for ISI due to the mechanical vibration and deformation of the fuel rod. Furthermore, the gap between the neighbor rods is only 3 mm. Including the thickness of the transducer, fuel rod deformation, and near-field region, there is a risk that the transducer might become stuck. It is necessary to find a feasible method to solve these problems.

The principle of the proposed inspection method is to detect whether there is water in the fuel rod by using an ultrasonic plate wave. The FA suffered from high-flow coolant with high temperature and high pressure in the reactor. When the fuel rod fails, the reactor coolant will enter the fuel rod from the damaged part due to the pressure difference inside and outside of the cladding.

#### 2.2.1. To Avoid the Influence of Near-Field Region

**Figure 2:**
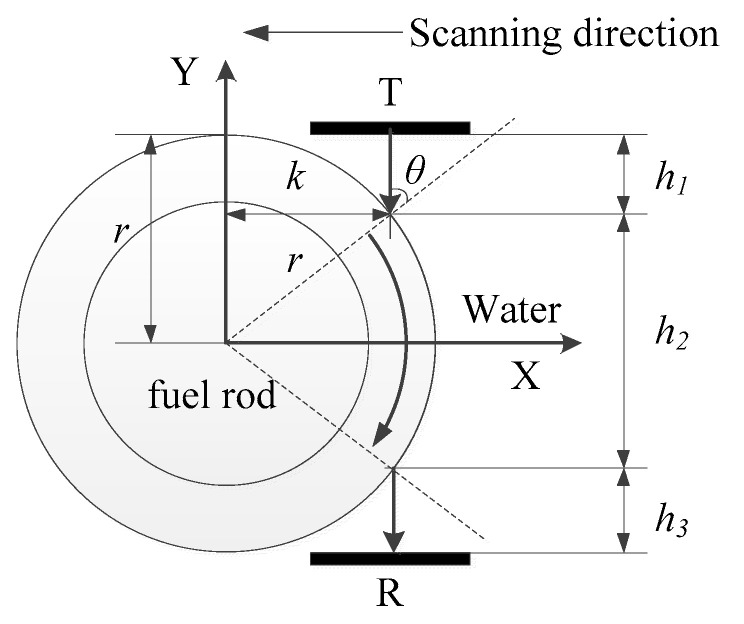
The diagram for avoiding influence of near-field region.

**Figure 3:**
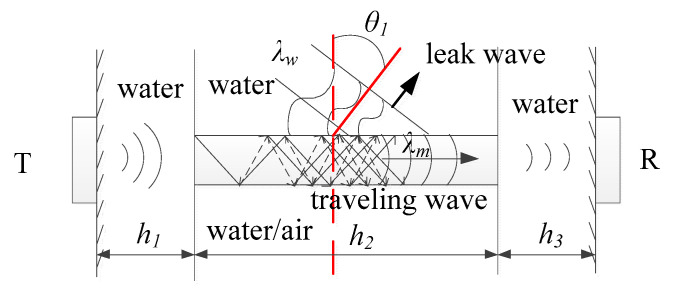
The principle of inspection.

In the proposed method, the wave propagation model without transducer liftoff is established, as shown in Figure 2. There are two T/R transducers. The longitudinal wave transmitted by the T transducer will pass through the water column *h*_1_ and incident into the rod at a certain angle θ. Then, the plate wave will be excited, which propagates along the circumferential rod *h*_2_. At last, the wave will be emitted into the water *h*_3_ and received by the R transducer. The ratio of the inner diameter to the outer diameter of the zirconium alloy rod is 88%. Therefore, it can be simplified to a thin plate model [17], as shown in Figure 3. Unfortunately, it involves three different media: air, water, and metal. The near-field region may be distributed in different media. The near-field region of the homogeneous medium can be calculated as:

(6) $$N=\frac{D_{s}^{2}}{4\lambda }$$

where *N* is length of near-field region, $D_{s}$ is diameter of transducer and λ is the wavelength in medium. The λ can be obtained as:

(7) $$\lambda =\frac{c}{f}$$

where *c* is sound velocity in the medium and *f* is the frequency of the transducer. Taking Equation (7) into Equation (6), Equation (6) can be rewritten as:

(8) $$N=\frac{D_{s}^{2}f}{4c}$$

According to Equation (8), the near-field region length $N_{1}$ in metal and water $N_{2}$ can be obtained as:

(9) $$N_{1}=\frac{D_{s}^{2}f}{4c_{1}}$$

(10) $$N_{2}=\frac{D_{s}^{2}f}{4c_{2}}$$

Equation (9) is divided by Equation (10), and the relationship between the metal and water near fields can be obtained as:

(11) $$\frac{N_{1}}{N_{2}}=\frac{c_{2}}{c_{1}}$$

where $c_{1}$ is sound velocity in metal, $c_{2}$ is sound velocity in water.

The propagation path of the sound wave is water–metal–water. The distance of different media is uniformly converted to metal media. Assume that the propagation distance of sound wave in water are $h_{1}$ and $h_{3}$, respectively, the propagation distance in the water can be converted to the distance in metal according to Equation (11). In other words, the distance in the water is equivalent to that in metal. Therefore, taking $h_{1}$ and $h_{3}$ into Equation (11), then the converted distance $l_{1}$, $\;l_{3}$ can be obtained as:

(12) $$\{\begin{array}{c} l_{1}=\frac{c_{2}}{c_{1}}h_{1}\\ l_{3}=\frac{c_{2}}{c_{1}}h_{3} \end{array}$$

Assume that the propagation distance in metal is $h_{2}$, then the total distance of the sound wave *P* passing through water–metal–water will be:

(13) $$P=\frac{c_{2}}{c_{1}}h_{1}+h_{2}+\frac{c_{2}}{c_{1}}h_{3}$$

where $h_{1}$,$\;h_{2}$,$\;h_{3}$ are the path of the wave through water–metal–water, respectively. Equation (13) demonstrates that the propagation distance of different media is equivalent to that of metal media. Thus, the remaining near-field region length can be obtained by subtracting the sound propagation distance *P* from the near-field length in metal. If the remaining near-field region length is above zero, that means the sound propagation distance is in the range of near-field region. In this situation, the inspection result is unreliable due to unstable sound pressure. The remaining near-field region length should meet the following condition:

(14) $$\frac{D_{s}^{2}}{4\lambda_{1}}-P=\frac{D_{s}^{2}f}{4c_{1}}-\frac{c_{2}}{c_{1}}h_{1}-h_{2}-\frac{c_{2}}{c_{1}}h_{3}\leq 0$$

According to our previous work [15] and Equation (5) with the geometric relationship, as shown in Figure 2, the distance can be obtained:

(15) $$\{\begin{array}{c} h_{1}=r\left(1-cos\theta \right)\\ h_{2}=\frac{\left(180-2\theta \right)\pi r}{180}\\ h_{3}=r\left(1-cos\theta \right) \end{array}$$

Taking Equation (15) into Equation (14), the following equation can be obtained:

(16) $$\frac{D_{s}^{2}f}{4c_{1}}-\frac{2c_{2}}{c_{1}}r\left(1-cos\theta \right)-\frac{\left(180-2cos\theta \right)\pi r}{180}\leq 0$$

From Equation (16), it can be seen that the plate wave excitation condition in Equation (5) is met, and the influence of the near-field region can be avoided by the selection of an appropriate frequency *f* according to the dispersion curve. 

#### 2.2.2. The Principle of Inspection

Unlike surface waves, plate waves are formed by the coupling of longitudinal waves parallel to the propagation direction and transverse waves perpendicular to the propagation direction. According to the principle that the ultrasonic propagation path is reversible, the leak wave will occur at the interface of metal–water, as shown in Figure 3. The calculation for the leak angle $\theta_{1}$ can be written as:

(17) $$\theta_{1}=arcsin\frac{\lambda_{w}}{\lambda_{m}}=arcsin\frac{v_{w}}{v_{p}}$$

where $\lambda_{w}$ is the wavelength in water and $\lambda_{m}$ is the wavelength in metal, the $v_{w}$ is wave velocity in water, the $v_{p}$ is the plate wave phase velocity.

Equation (17) describes the degree of wave leakage. The greater the $v_{w}/v_{p}$, the greater the attenuation. In contrast, the leak wave will not occur at the metal–air interface due to the big acoustic impedance difference between the air and metal.

Therefore, if there is no water in the cladding, the wave will reflect completely at the zirconium–air interface with low attenuation. However, if the fuel rod is failed, the water in the cladding provides a path for the leakage of plate wave energy. The R transducer will receive a lower amplitude signal. Furthermore, there is a linear relationship between amplitude and attenuation due to being far away from the near-field region. Thus, the failed rod can be inspected by amplitude.

#### 2.2.3. The proposed algorithm

**Figure 4:**
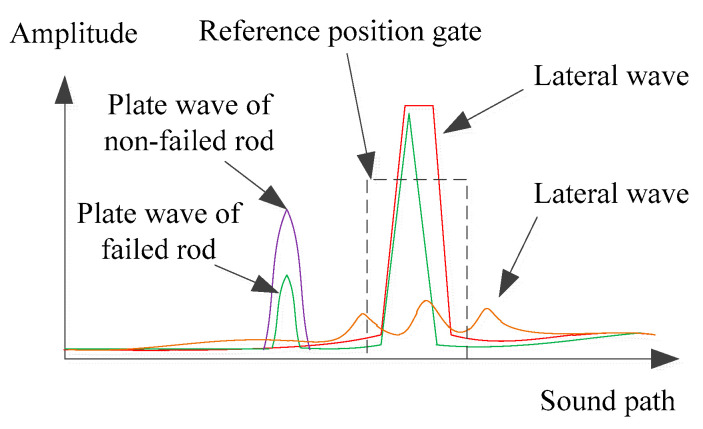
The A-scan signal during scanning process.

The scanning direction is from right to left. Since the tube is symmetrical along the Y-direction, the signal in the scanning process is also axisymmetric. Therefore, taking the right side of the rod into consideration, the incident angle of ultrasonic wave *θ* will change from 90° to 0°. The A-scan signal in the scanning process can be divided into 3 parts. At first, the fuel rod is out of the ultrasound range. The transmitted wave will be received by the receiving transducer completely. The signal at this time has been taken as the reference position called lateral wave as the red line, as shown in Figure 4. Secondly, the plate wave is excited as the transducer’s movement. At this moment, the received signal is complicated. Because of that, not only the plate wave that passes through the rod can be received, but also the wave passing through the water column. In addition, the different wave modes will also be received. In addition, the amplitude of the plate wave passing through the failed rod is lower than that non-failed rod, as the green and purple lines in Figure 4 show. At last, when the transducer moves to the center of the tube, most of the transmitted wave will be blocked. Therefore, the lateral wave was the lowest, as the orange line shows in Figure 4.

**Figure 5:**
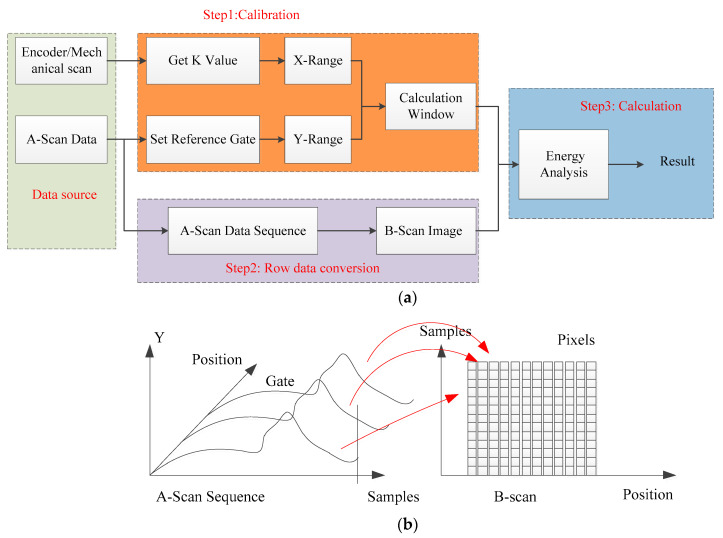
(**a**) The proposed processing method; (**b**) the principle of B-scan image.

Due to the overlap of wave modes, the actual signal is cluttered. It is difficult to distinguish whether it is a plate wave or a scattered signal because of scanning movement. In order to solve this problem, the signal energy evaluation method based on the B-scan image proposed for an in-service fuel rod failure inspection, as shown in Figure 5a. The proposed architecture of the working procedure can be divided into three steps. There are two kinds of data sources; one is an A-scan sequence and another is an encoder pulse. During scanning, the motor drives the encoder to rotate and send out pulse signals. The encoder pulses provide the mechanical position of transducers since the pulses can be calculated to distance. Therefore, the A-scan waves at different positions can be recorded at the same time. In other words, the A-scan sequence corresponds to the scanning position.

In order to verify the correctness of distance calculation, the first step is to calibrate ratio *K* between the actual moving distance and the encoder pulse. Ratio *K* can be calculated as:

(18) K=d/n

where *d* is the measured distance and n is the acquired count of encoder pulses.

After that, take the radius of the rod as the size of the calculation window in the horizontal direction. After that, the reference point is set at the lateral wave by a gate when the fuel rod is outside of the ultrasonic range. The time of flight of the plate wave before the reference point is recorded as the size of the calculation window in the vertical direction. Building the calculation window as a sliding window in a B-scan image to calculate echo energy. The above process is called calibration.

The second step is to convert the A-scan waves into a B-scan image. As mentioned before, since the A-scan sequence corresponds to the scanning position, the B-scan image can be obtained by arranging the A-scan signal sequence along the mechanical position and replacing the amplitude with color. The principle of the B-scan image is shown in Figure 5b.

The third step is to calculate the echo energy on B-scan image by using the sliding window. The echo energy on B-scan image is calculated as follows.

(19) $$\delta =\sqrt{\frac{1}{M\times N}\sum_{i=1}^{M}\sum_{j=1}^{N}{\left(P\left(i,j\right)-u\right)}^{2}}$$

where M×N is the size of sliding window, P(i,j) is the pixel values of row *i* and column *j* on the B-scan image, and u is the mean. Here the u is zero due to the echo amplitude compared with zero.

## 3. Simulation and Experiments Setup

The fuel rod is a cladding tube made of zirconium alloy. Commonly, the outer diameter and wall thickness are 9.5 mm and 0.57 mm, respectively, in Chinese PWR nuclear power plants. Each FA consists of 17 × 17 fuel rods called AFA 3G. The outer diameter of a fuel rod is 9.5 mm, and the center distance between the fuel rods is 12.6 mm.

**Figure 6:**
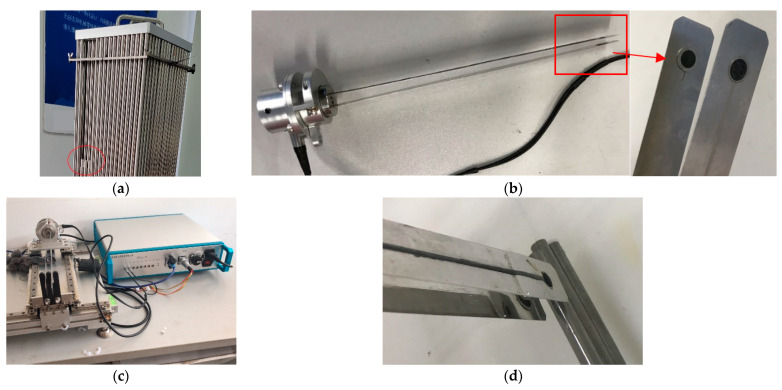
(**a**) The FA specimen; (**b**) the fabricated transducers; (**c**) the mechanical device and instrument; (**d**) the transducers are coupling with rods.

In order to validate the proposed technique, taking three rods with a diameter of 9.5 mm and a wall thickness of 0.57 mm as an example, the simulation and experiment were carried out. The specimen is made by the manufacturers of zirconium alloy cladding rods which are used for the FA. The rods are filled with dry sand or wet sand to simulate normal fuel rods or failed fuel rods, respectively. There are two kinds of rods—non-failed or failed fuel rods. There were17 fuel rods in total for testing. Three of them were failed rods, as shown in Figure 6a.

A 5 MHz frequency and a 6 mm sized transducer was selected for inspection. According to Equation (5), Equation (16), and the dispersion curve, the remaining near-field region length was −14.29 mm. That means the distance between the T/R transducers is longer than the near-field region length. Therefore, the signal received by the R transducer is linear. More detail is shown in Table 1. The T/R transducers have the same acoustic parameters mounted on two thin metal sheets. The thickness of the metal sheets is only 1 mm in order to insert in the gap of FA. The transducers are fabricated for testing, as shown in Figure 6b.

The simulation was calculated using the CIVA software developed by CEA (French Atomic Energy Commission) software. At the same time, the mechanical devices and transducers were fabricated for testing. With the device, the transducers can be moved along the x–y–z directions arbitrarily with calibration. In the experiment, the testing rods were submerged in water, and the transducers were moving to inspect the specimens. As mentioned before, after calibration, the mechanical movement drives the encoder to rotate to obtain the displacement in real-time. The ultrasonic instrument acquires displacement and echoes simultaneously. Then the A-scan sequence can be transformed into a B-scan image by using a palette.

The experiment has also been carried out to validate the proposed method. The ultrasound acquisition instrument with a working frequency from 0.2 MHz to 20 MHz is shown in Figure 6c. The transducer is excited by a square wave with a 200 V voltage in the experiment setup. The ultrasonic signal is acquired with a 100 MHz frequency. The rods are immersed in water and coupled with transducers in the experiment, as shown in Figure 6d.

## 4. Results and Discussion

**Figure 7:**
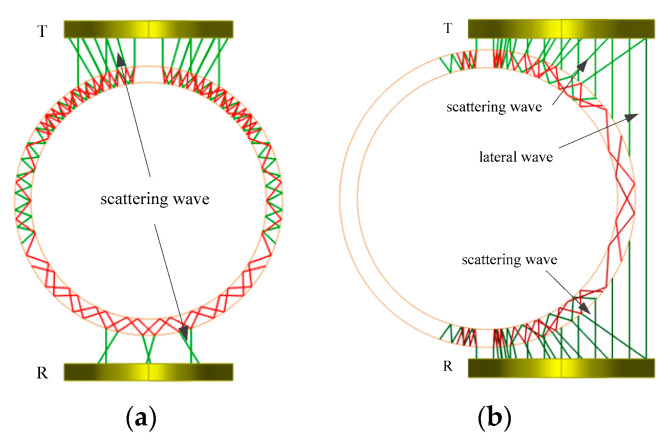
(**a**) The wave propagation when the rod is in the center of the transducers; (**b**) the wave propagation when a part of the rod enters the transducer.

For the experimental specimen, the sound field and propagation simulation of the transducer has been carried out. The wave propagation of the probe at different positions is shown in Figure 7a and Figure 7b, respectively. The green lines in Figure 7 represent for longitudinal wave, and the red lines show the wave transformation from the longitudinal wave. Figure 7a shows the wave propagation when the rod is in the center of the transducers. It demonstrates that most of the ultrasonic waves propagate on the tube wall, and only a small part of the energy can be received by the R transducer. On the other hand, a part of the rod enters the transducer when the plate wave is excited, more waves can be received, as shown in Figure 7b. It demonstrates that part of the transmitted wave directly passes through the water column to the R transducer, which is called a lateral wave. The other part of the received wave is the scattering wave at the zirconium–water interface.

**Figure 8:**
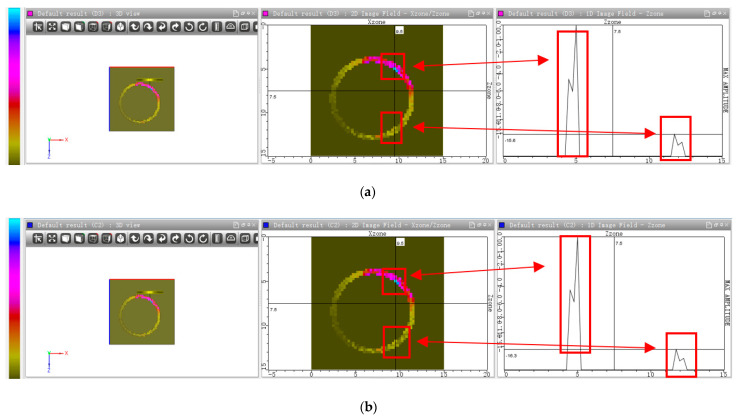
(**a**) The sound field with normal fuel rod; (**b**) the sound field with failed fuel rod.

In addition, the sound field simulation was also carried out at the position where the plate wave was excited, as shown in Figure 8. It is the sound pressure distribution of the transmitted sound field in the rod. The color presents the amplitude of sound pressure, as shown by the color palette. The bluer the color, the greater the amplitude. The incident point is symmetrical to the exit point due to the symmetry of the rod. The red rectangles represent the incident point and its sound pressure and the exit point and its sound pressure, respectively. The 1D sound field curve is different from the A-Scan curve. It represents the sound pressure along the z direction.

Figure 8a shows the sound field in the rod and the corresponding 1D sound field with a normal fuel rod. The wave amplitude at the exit point is −15.6 dB. Figure 8b shows the sound field in the rod and the corresponding 1D image field with failed fuel rod. It can be easily seen that the wave amplitude at the exit point is −16.3 dB. The amplitude of the failed rod decreases by 0.7 dB.

**Figure 9:**
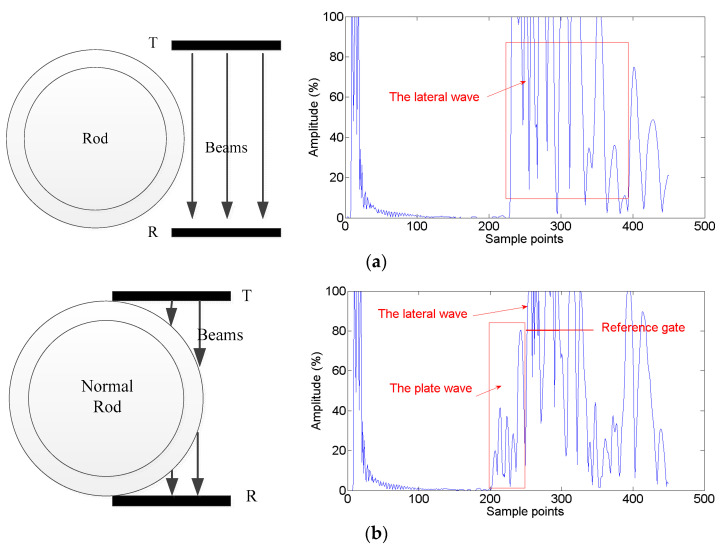

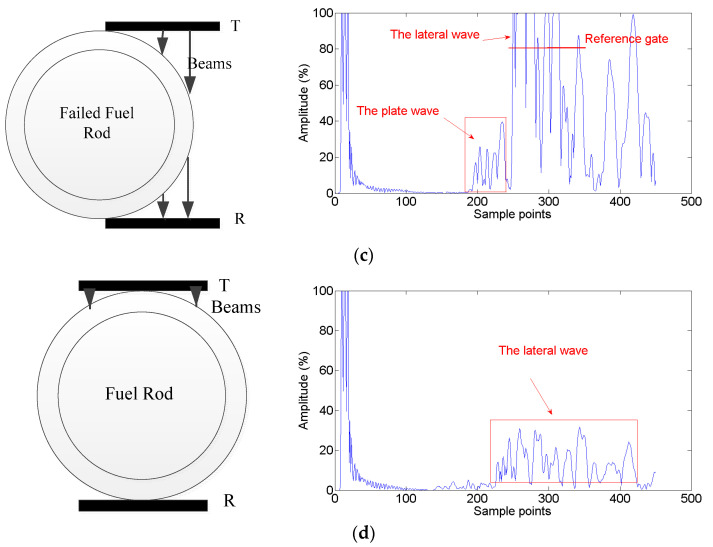
(**a**) The signal when the rod is out of ultrasound range; (**b**) the signal of normal fuel rod when part of the rod is in ultrasound range; (**c**) the signal of failed fuel rod when part of the rod is in ultrasound range;(**d**) the signal when the rod is in the center of the T/R transducers.

**Figure 10:**
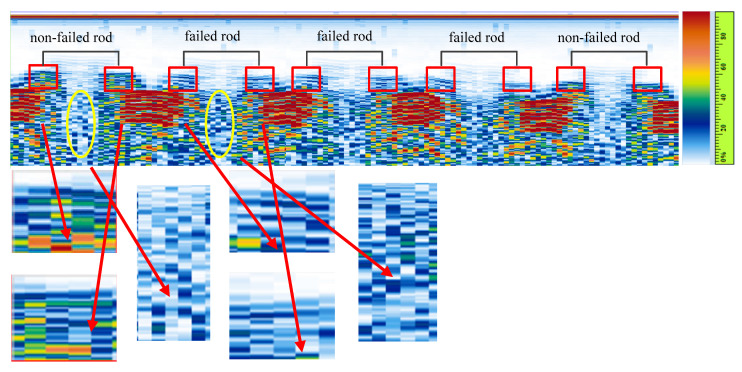
The B-scan image with sliding window.

In order to validate the proposed method and simulation result, the experiment has been carried out. The steps of the processing are listed as follows:

(1) Calibrate the mechanical *K* value and set the reference gate.
(2) The A-scan raw data are acquired from the instrument, as shown in Figure 9.
(3) The sliding window is created based on the result of calibration.
(4) The A-scan sequence is converted into a B-scan image, as shown in Figure 10.
(5) Finally, according to Equation (19), the energy curve of the plate wave can be obtained by sliding the window on the B-scan image.

Figure 9a–d shows the signals of the three stages of the scanning process as described before. When the rod was out of the ultrasound range, the transmitted wave was received through the water column. Therefore, only the lateral wave signal can be seen, as shown in Figure 9a. As the transducers are scanning, part of the rod is in the ultrasound range. In this situation, not only the lateral wave but also the plate wave can be received by the R transducer. It can be clearly seen that the plate wave is in front of the lateral wave due to ultrasound travelling faster in metal than in water, as shown in Figure 9b,c. When the rod is in the center of the T/R transducers, the plate wave excitation condition does not meet Equation (5). Therefore, there is no plate wave, and only a small part of the wave can be received. The amplitude of the lateral wave is low, as shown in Figure 9d. In particular, Figure 9b,c show the signal of the normal fuel rod and failed fuel rod, respectively. It can be clearly seen that the plate waves of different modes are overlapped to multiple peaks. The average amplitude of the normal fuel rod is higher than that of the failed fuel rod. The max amplitudes of the normal fuel rod and the failed rod are 80% and 40%, respectively. It is approximately 6 dB higher. It is apparent that the plate wave is far away from the near-field region due to the sufficient distance and the plate wave in the T/R mode.

Figure 10 shows the B-scan image converted from the A-scan sequence. It can be clearly seen that the signal of the fuel rod is composed of left and right parts, respectively. The sliding window is shown on the B-scan image as red rectangles. The yellow ellipse shows the signal when the rod is at the center of the T/R transducers. From the color palette, we can see that the redder the color, the higher the amplitude. Therefore, the pixel color in the red rectangle is able to demonstrate the average energy. The more red or orange color pixels, the higher the average energy. It is apparent that the average energy of the non-failed rod is higher than that of the failed rod. Meanwhile, the average energy in the yellow ellipse of the non-failed rod is lower than that of the failed rod. The size of the calibrated sliding window is 140 × 85 pixels. According to Equation (17), the plate wave energy of the failed fuel rod is 3.1913 × 10³ and 3.5308 × 10³. By comparison, the plate wave energy of the normal fuel rod is much higher, which is 4.1068 × 10³ and 4.4032 × 10³, respectively. The detailed results of all fuel rods are shown in Table 2. All of the failed fuel rods can be detected. The detection rate of the failed fuel rods can reach 100%. Compared with the previous work [3], the detection rate increased by 7%. The comparison is shown in Table 3.

In conclusion, the experimental result is consistent with the simulation result. Thus, the correctness of the proposed failure inspection method based on the plate wave principle is proved.

## 5. Conclusions and Future Work

In this paper, a novel fuel rod failure inspection method has been proposed by using ultrasonic plate waves. A deep analysis of wave propagation and T/R signal has been presented during the scanning process. The verification experiments were conducted to validate the effectiveness of failed fuel rod detection. Several conclusions can be drawn: (i). the influence of the near-field region can be avoided for ISI by using the proposed method. (ii) the proposed system inspection frequency is only 5 MHz, so it is independent and is a wide-bandwidth and high-performance instrument. (iii). the proposed system enables us to inspect all the failed fuel rods in ISI. Future work will carry out the FA deformation measurement with the deep learning method.

## Figures and Tables

**Table 1:** The acoustic parameters calculated by proposed method.

Frequency	Shape	Size	Near-Field Region	Distance	Remaining Near-Field Region
5 MHz	circle	Φ6	9.6 mm	23.89 mm	−14.29 mm

**Table 2:** The results of the proposed method.

Samples	Left Part	Right Part	Ground Truth	Proposed Method
1	4.3417 × 10³	4.5467 × 10³	non-failed	non-failed
2	4.4861 × 10³	4.4369 × 10³	non-failed	non-failed
3	3.1913 × 10³	3.5308 × 10³	failed	failed
4	3.3125 × 10³	3.4235 × 10³	failed	failed
5	3.2957 × 10³	3.4621 × 10³	failed	failed
6	4.1068 × 10³	4.4032 × 10³	non-failed	non-failed
7	4.6394 × 10³	4.5393 × 10³	non-failed	non-failed
8	4.2454 × 10³	4.3478 × 10³	non-failed	non-failed
9	4.4921 × 10³	4.2879 × 10³	non-failed	non-failed
10	4.5265 × 10³	4.4766 × 10³	non-failed	non-failed
11	4.3577 × 10³	4.2398 × 10³	non-failed	non-failed
12	4.5231 × 10³	4.3433 × 10³	non-failed	non-failed
13	4.2463 × 10³	4.4513 × 10³	non-failed	non-failed
14	4.4254 × 10³	4.6833 × 10³	non-failed	non-failed
15	4.5286 × 10³	4.3724 × 10³	non-failed	non-failed
16	4.2985 × 10³	4.1284 × 10³	non-failed	non-failed
17	4.4943 × 10³	4.2652 × 10³	non-failed	non-failed

**Table 3:** The comparation of proposed method.

Method	Accurate	Liftoff
bulk wave	93%	1 mm
plate wave	100%	0 mm

## Data Availability

Not applicable.

## Nomenclature

FA	Fuel assembly
NDT	Non-destructive testing
VT	Visual testing
EC	Eddy Current
UT	Ultrasound testing
PWR	Pressurized water reactor
AFA 3G	Advanced fuel assembly 3 Generation
ISI	In-service inspection
